# Biochemical Discrimination between Selenium and Sulfur 2: Mechanistic Investigation of the Selenium Specificity of Human Selenocysteine Lyase

**DOI:** 10.1371/journal.pone.0030528

**Published:** 2012-01-26

**Authors:** Ann-Louise Johansson, Ruairi Collins, Elias S. J. Arnér, Peter Brzezinski, Martin Högbom

**Affiliations:** 1 Arrhenius Laboratories for Natural Sciences C4, Stockholm Center for Biomembrane Research, Department of Biochemistry and Biophysics, Stockholm University, Stockholm, Sweden; 2 Structural Genomics Consortium, Department of Medical Biochemistry and Biophysics, Karolinska Institute, Stockholm, Sweden; 3 Division of Biochemistry, Department of Medical Biochemistry and Biophysics, Karolinska Institute, Stockholm, Sweden; University of Queensland, Australia

## Abstract

Selenium is an essential trace element incorporated into selenoproteins as selenocysteine. Selenocysteine (Sec) lyases (SCLs) and cysteine (Cys) desulfurases (CDs) catalyze the removal of selenium or sulfur from Sec or Cys, respectively, and generally accept both substrates. Intriguingly, human SCL (hSCL) is specific for Sec even though the only difference between Sec and Cys is a single chalcogen atom.

The crystal structure of hSCL was recently determined and gain-of-function protein variants that also could accept Cys as substrate were identified. To obtain mechanistic insight into the chemical basis for its substrate discrimination, we here report time-resolved spectroscopic studies comparing the reactions of the Sec-specific wild-type hSCL and the gain-of-function D146K/H389T variant, when given Cys as a substrate. The data are interpreted in light of other studies of SCL/CD enzymes and offer mechanistic insight into the function of the wild-type enzyme. Based on these results and previously available data we propose a reaction mechanism whereby the Sec over Cys specificity is achieved using a combination of chemical and physico-mechanical control mechanisms.

## Introduction

Selenium is the only trace element found in proteins that is directly genetically encoded. The semi-metal is incorporated into selenoproteins in the form of selenocysteine (Sec) by a co-translational process using an intricate translation mechinery that redefines specific UGA codons to encode Sec [1], [2]. Selenium can also be severely toxic because of the high chemical reactivity of metabolites such as selenite and hydrogen selenide [3], [4], [5]. Thus it is vital to both have adequate selenium intake and to develop means for tight control of the selenium metabolism. Sec ingested in the diet is not directly loaded on its tRNA for subsequent incorporation in selenoproteins. Instead, Sec is first degraded by Sec lyase (SCL) that can provide the selenide precursor for synthesis of selenophosphate, subsequently used for conversion of tRNA^Sec[Ser]^ to tRNA^Sec[Sec]^ [6], [7], [8], [9], [10], [11], [12].

Sec lyases (SCLs) and Cys desulfurases (CDs) catalyze the removal of selenium or sulfur from Sec or Cys and generally act on both substrates. Importantly, however, human SCL (hSCL) as well as SCLs from other higher eukaryotes are specific for Sec and do not accept the sulfur analogue Cys as substrate. Instead, hSCL is inhibited by addition of excess Cys [9], [12], [13], [14], [15].

**Figure 1:**
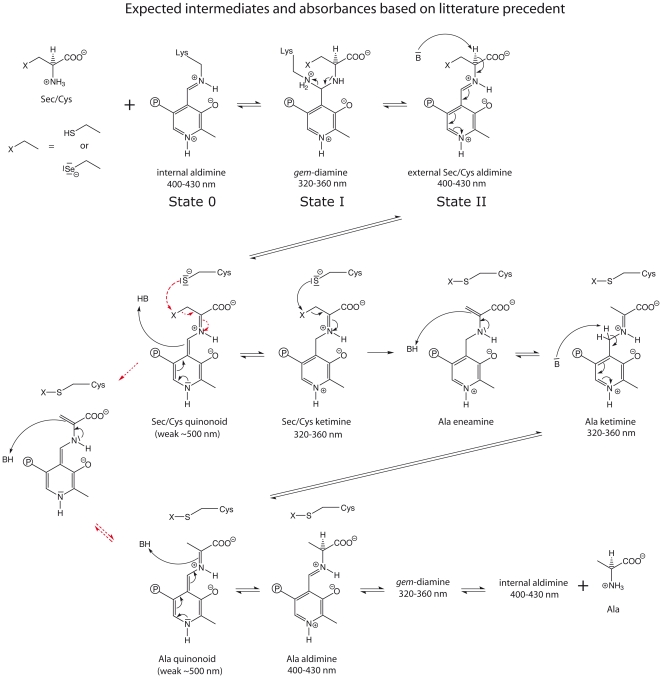
Consensus mechanism in SCL/CD enzymes and related systems, a variation of the mechanism, proposed in some studies is indicated by red dotted arrows [10], [15], [16], [17], [18], [19], [20], [21], [22], [23], [24]. Shown in the scheme are the Sec/Cys substrates, the presumed interaction with the PLP cofactor, and the active site Cys residue accepting either sulfur or selenium. The intermediate species denoted as “State 0”, “State I” and “State II” are further discussed in the text.

The enzymatic mechanism for CD enzymes was originally delineated by Zheng et al. This has since served as a model for several mechanistic proposals for SCL and CD proteins resulting in a consensus mechanism (Fig. 1), with some studies suggesting a variant of the mechanism in that the SH or Se^−^ is eliminated directly from the Sec/Cys quinonoid intermediate (red dotted arrows in Fig. 1) [10], [15], [16], [17], [18], [19], [20], [21], [22], [23], [24].

Still, the structural and chemical basis for the important selenium specificity of eukaryotic SCLs remains unclear. A recent study of SCL from rat (rSCL) reported slightly different binding modes for Cys and the Sec substrate analogue selenopropionate and suggested this to be the basis for specificity. Cys was reported to reversibly form a nonproductive adduct with rSCL while selenopropionate bound in two different conformations [25]. However, the guiding mechanism and whether the binding is influenced by the lack of the amine on the Sec substrate analogue used remains an open question.

In an accompanying study, using a structure-guided bioinformatic approach, we produced gain-of-function protein variants of hSCL that also show CD activity. Among the protein variants tested, a D146K variation was necessary and sufficient to obtain CD activity in hSCL [26]. The aim of this study is to benefit from these results to gain further insight into the mechanism of SCL/CD enzymes and the chemical basis for selenium specificity in hSCL. Here we report time-resolved spectroscopic characterization of the selenium-specific wild-type (WT) hSCL in comparison with the D146K/H389T protein variant that shows gain-of-function for Cys cleavage. The double mutant was choosen for study because it showed slightly higher activity than the D146K single mutant [26]. The data indicate that the wild type and active variant proteins behave similarly in the early steps of the reaction while differences are observed in later stages. Based on these results and previously available data, we hypothesize a reaction mechanism including a chemical specificity step that provides the selenium specificity of hSCL.

## Results

### Gain-of-function variants

We found that in contrast to wild type hSCL, which has a very low activity with Cys as substrate, a mutation of D146 into Lys resulted in a gain-of-function variant with both Sec lyase and Cys desulfurase activity. The structural determinants for this activity were studied in an accompanying paper [26]. Here, we wished to provide further insight into the chemical basis for this specificity using time-resolved spectroscopy. The double mutant D146K/H389T was choosen for study because it showed slightly higher activity with Cys than the D146K single mutant [26].

#### Time-resolved UV/Vis spectroscopy

**Figure 2:**
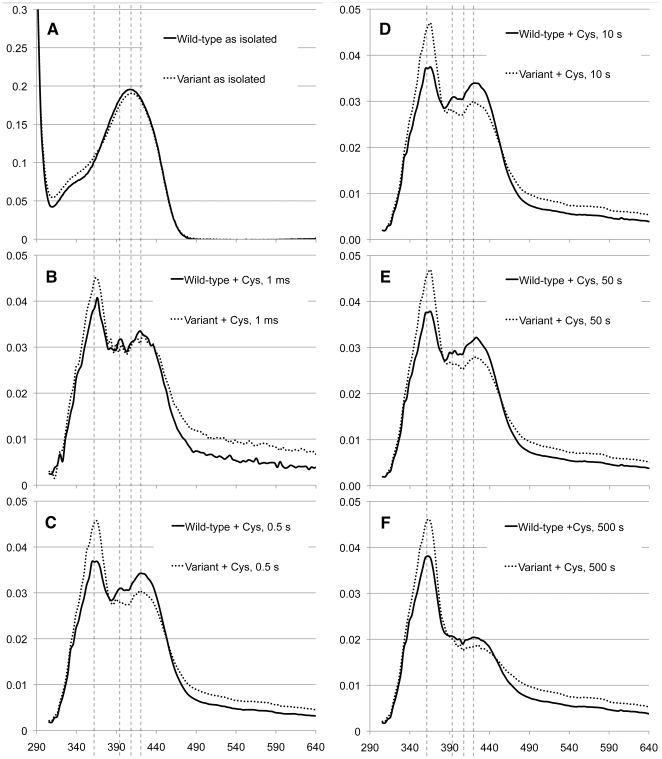
Spectroscopic data for the wild-type and the D146K/H389T variant proteins. A) Spectra of as-isolated proteins taken in a scanning spectrophotometer. B–F) Diode-array stopped flow spectra 0.001, 0.5, 10, 50 and 500 sec after addition of 10 mM Cys. Reference lines are drawn at 360, 395, 410 and 420 nm.

**Figure 3:**
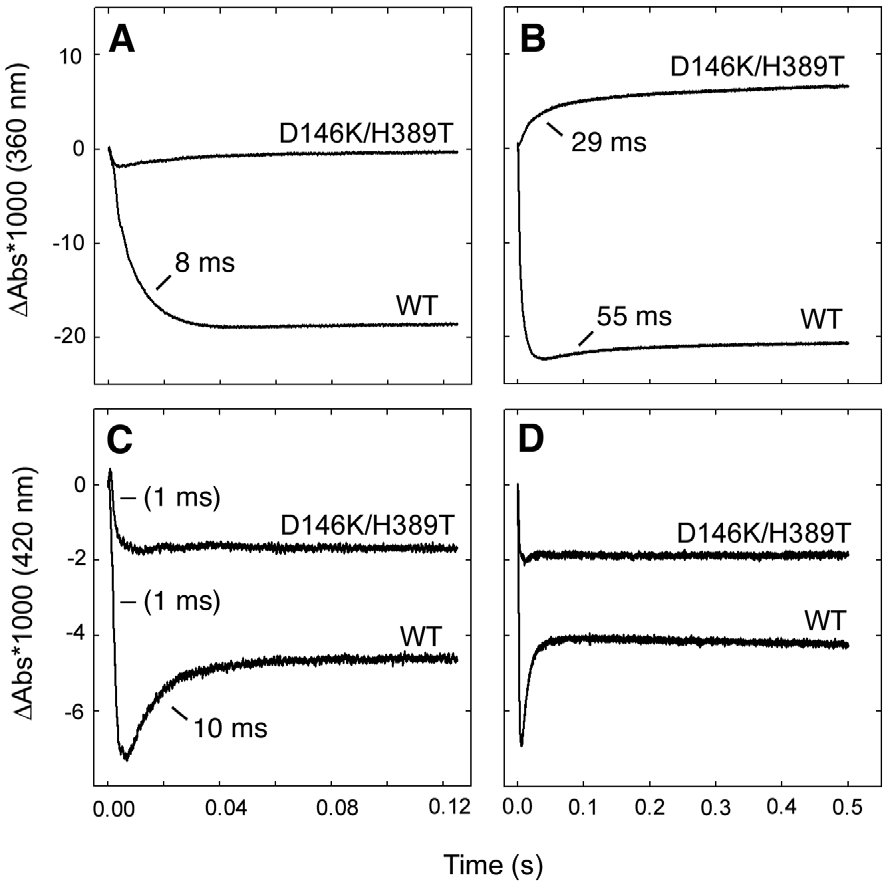
Absorbance difference traces at 360 nm (A, B) and 420 nm (C, D) upon addition of Cys as a function of time for the wild-type and D146K/H389T variant proteins. Panels (A, C) 0–125 ms, panels (B, D) same wavelength up to 500 ms. Note that these traces show the differences in absorbance after mixing. The absorbance at time = 0 was set to zero independently of the actual value, which means that the absolute absorbance values at different wavelengths can not be directly compared. Time constants, determed from fits with a sum of exponential functions, are also given.

To investigate how the gain-of-function substitution influenced the mechanism, and thus the chemical background to the selenium specificity of the wild-type protein, we performed spectral analyses and stopped-flow UV/Vis spectroscopic investigations of the reaction of the wild-type and D146K/H389T variant proteins using Cys as substrate (Fig. 2A–F and Fig. 3 A–D). The UV-visible spectrum of the as isolated (in 120 mM Tris pH 8.5 and 2 mM TCEP) wild-type and variant proteins showed similar spectra with an absorption peak at ∼410 nm with only a slight shoulder in the 300–350 nm region (Fig. 2A), which was very similar to what has been observed in other NifS homologs [15], [27]. This reveals that the Shiff base linking the PLP cofactor to the protein exists mainly in the protonated, ketoenamine, form of the internal aldimine species (Fig. 1, “state 0”) [18], [28].

The enzyme solution was mixed with Cys to final concentrations of 20 µM enzyme and 10 mM Cys in a stopped-flow apparatus. The first spectrum, obtained immediately after mixing (∼1 ms mixing time), displayed major peaks at ∼360 nm and 420 nm and a minor contribution at ∼395 nm with both proteins behaving virtually identical on this short time scale (Fig. 2B). At 0.5 sec the wild type spectrum remained basically unchanged but with a slight net decline of the 360 nm peak and accumulation of the 420 nm peak. The variant protein, on the other hand, showed a decline of the 420 nm peak (Fig. 2C). After 0.5 s both spectra remained qualitatively unchanged in the minute regime (Fig. 2D,E), but displaying a slow decline of the 420 nm peak over several minutes. After long incubation (500 s) both systems appeared to have reached quasi-steady state with absorbance contributions from all states of the reaction in their respective equilibrium concentrations.

To determine the kinetics of the specific reaction steps, we measured the time-dependent absorbance changes for both protein variants up to 500 ms (Fig. 3). Note that these traces show the differences in absorbance after mixing. The absorbance at time = 0 was set to zero independently of the actual value, which means that the absolute absorbance values at different wavelengths can not be directly compared. Time constants obtained by fitting the data are also shown. The very rapid decay in absorbance at 420 nm coincides in time with the mixing time of the instrument, estimated at ∼1 ms, and therefore the time constant of the corresponding reaction could only be estimated at ≤1 ms. For the wild type protein, three distinct phases were observed, firstly a very rapid phase (≤1 ms) indicated by a decline in the 420 trace. Next, the absorbance increased at 420 nm and decreased at 360 nm with a time constant of ∼10 ms. Finally, a phase with a time constant estimated at >50 ms was observed as a small increase at 360 nm. The traces for the variant protein show an initial rapid (≤1 ms) decrease at 420 nm, followed in time by an increase in absorbance at 360 nm with a time constant of 29 ms.

**Figure 4:**
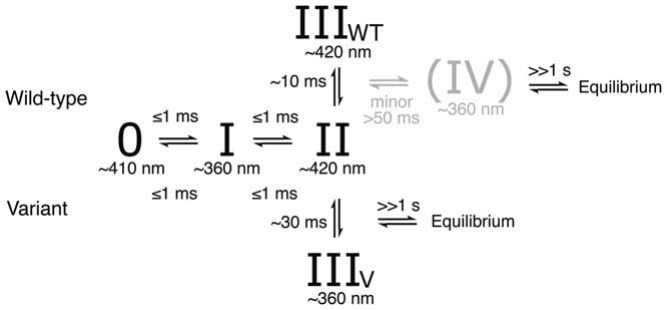
Model describing the observed transitions in the reaction of WT hSCL and the D146K/H389T variant protein with excess cysteine as inferred from in the combined spectroscopic data. Discussion of the possible nature of “State 0”, “State I” and “State II” are found in the text and also identified in Figure 1.

The combined spectroscopic data can be described by the model in figure 4. The transition from the 410 nm absorbance of the as-isolated protein (“State 0”, Fig. 4) to the 360 nm (“State I”, Fig. 4) and 420 nm (“State II”, Fig. 4) absorbances dominating at 1 ms must be very fast and is not observed in the absorbance difference traces. We conclude that this phase was rapid and over within the mixing time of the instrument (∼1 ms) for both proteins. A decay of the 420 nm absorbance is however observed just after t = 0 for both proteins (Fig. 3C). Following these very rapid phases, an absorbance decay at 360 nm was observed in the wild-type protein concomitant with an increase in absorbace at 420 nm with a time constant of ∼10 ms (Fig. 3A, C). In the D146K/H389T variant enzyme this phase was not observed, still, the 420 nm absorbing species is present with similar amplitude in both spectra at 1 ms (Fig. 2B). The most likely interpretation of this data is that the 420 nm absorbance observed in the wild-type protein is a result of more than one species of which one is formed, and decays, fast (as in the variant protein) and one is slower in its formation and accumulates with a time constant of ∼10 ms. Due to the qualitatively similar spectra we presume that the same early intermediate species were produced in both proteins. After the initial phases, we observe a phase in the D146K/H389T protein with a time constant of ∼29 ms, seen as an increase in absorbance at 360 nm (Fig. 3B). Indication of a similar absorbance increase can also be observed in the wild-type enzyme. It was difficult to accurately estimate the time constant of this phase, but we conclude that it is slower (>50 ms) and, based on the smaller increase in absorbance, appears to represent only a fraction of the sample.

The difference spectra at longer timepoints 0.5, 10, 50 and 500 sec (Fig. 2C–F) reveal that there is at least one more phase observed, characterized by a very slow drop in the absorbance at 420 nm, until the systems reach equilibrium.

## Discussion

The properties of the initial species absorbing at 360 nm (“State I”, Fig. 4), together with its rapid formation (cf. Fig. 2A and 2B), make the substrate gem-diamine species (Fig. 1) the most likely explanation for this absorbance [17], [18], [19], [24], [29], [30]. Based on the general reaction scheme, the rapidly forming 420 nm absorbing species in both systems (Fig. 2B) most likely result from the external Cys-aldimine (“State II”, Fig. 1). The minor peak at 390–395 nm should be consistent with a small amount of free PLP in solution, possibly liberated from the enzyme. After the initial very fast phases, we observe an accumulation of the absorbance at 420 nm in the wild type protein with a time constant of ∼10 ms (Fig. 3C, “State III_wt_” in Fig. 4). The identity of this species is difficut to assign because several intermediates are expected to absorb at this wavelength, moreover, it may also represent a species that is not part of the normal catalytic pathway. After this phase we see a gradual recovery of the absorbance at 360 nm with a time constant of ∼30 ms in the variant enzyme and >50 ms in the wild type protein. At this stage the spectra are expected to contain contributions from several intermediate states that also absorb at similar wavelengths. Finally, we observe a very slow phase, characterized by a decrease in absorbance at 360 nm, until the systems reach equilibrium. The last spectra at 500 s should represent both systems in equilibrium with excess cysteine. Here, all states of the protein contribute to the spectrum based on their relative abundance in the sample. It is also possible that various other species, such as thiol adducts observed in the rat enzyme, which are not part of the direct catalytic pathway but known to form in these systems contribute to the spectra [24], [25], [29]. Even if such dead-end substrate-enzyme species contribute statically to the spectra this would not influence the time constants observed in the transition between different species of the pathway. The time constants of the observed phases are also reasonable when compared with previously published catalytic parameters for SCL/CD enzymes [12], [13], [15], [24].

### Mechanism of selenium specificity

Previous investigations of the mechanism of SCL/CD enzymes and related systems show that the first step is the binding of substrate to form the PLP external aldimine, followed by deprotonation of the substrate. Eventually, a PLP-substrate intermediate is attacked by a deprotonated active site Cys, producing the protein-bound persulfide or sulfoselenide (Fig. 1) [10], [15], [16], [17], [18], [19], [20], [21], [22], [23], [24]. The spectroscopic data with excess Cys presented here is in good agreement with previous studies and the proposed general mechanism presented in figure 1. Still, because of the complexity of the reaction, with several different intermediates absorbing at similar wavelengths, it is not trivial to conclusively assign where the reaction halts in the wild type enzyme, explaining its low turnover with Cys. However, it appears to be in a step subsequent to formation of the external Cys-aldimine intermediate and we conclude that a very long-living absorbance at 420 nm accumulates to a larger extent in the inactive wild type protein, compared to the D146K/H389T variant having a gain-of-function allowing it to use Cys as well as Sec [26].

A hypothetical explanation for the Sec specificity of hSCL could be that the reaction with Cys is a single-turnover reaction, producing a dead-end protein adduct. The stopped-flow data as such can not allow a strict distinction of whether or not a single-turnover reaction has occurred in the WT protein. Also, single turnover experiments were not possible because of the high K_m_ of SCL for free Sec/Cys [12], [13]. However, we find this alternative unlikely as the basis for specificity because this implies that Cys would function as a suicide substrate. This does not seem to be the case based on published activity data [9], [12], [13] and moreover, it would mean that any newly synthesized protein in the cell would immediately be inactivated by reaction with Cys, that is much more abundant than Sec.

An important clue as to the chemical background for the Sec-over-Cys specificity of hSCL comes from a study of the Cys desulfurase from *Synechocystis* sp. PCC 6803 (*Sy*CD) and its inactive C325A variant [29] (C325 in *Sy*CD corresponds to C388 in hSCL). Upon addition of Cys, these proteins show a behavior highly similar to what we observe for the D146K/H389T variant and wild type hSCL, respectively. In the C325A variant of *Sy*CD, an absorbance profile with maxima of rougly equal absorbances at ∼340 and ∼417 nm develop within 80 ms and the corresponding state is stable in the minute regime. Based on a number of different observations, this species is assigned as the Cys aldimine and Cys ketimine in equilibrium, unable to proceed to substrate desulfuration because of the lack of the attacking cysteinyl sidechain [29]. In WT *Sy*CD, on the other hand, the spectrum is very similar to the hSCL active variant protein and dominated by the low wavelength absorbance. Taken together, the spectroscopic data and the observation that WT hSCL behaves similarly to the *Sy*CD C325A variant suggests that the lack of activity results from that C388 in WT hSCL is, for some reason, incapable of attacking the PLP-Cys intermediate.

The D146K/H389T variant showed slightly increased activity compared to the D146K single mutant [26]. H389 directly follows C388 in the sequence and also directly interacts with both D146 and C388 in the structure; it is thus not surprising that this variation may influence the activity slightly. However, as shown in the accompanying paper [26], the D146K variation was necessary and sufficient to provide CD activity to hSCL and it is clear that this is the key change to obtain CD activity. How may then D146 influence the attacking step to prevent desulfuration, while it becomes possible in the D146K variant protein? Our current working hypothesis and reasoning is described as follows.

**Figure 5:**
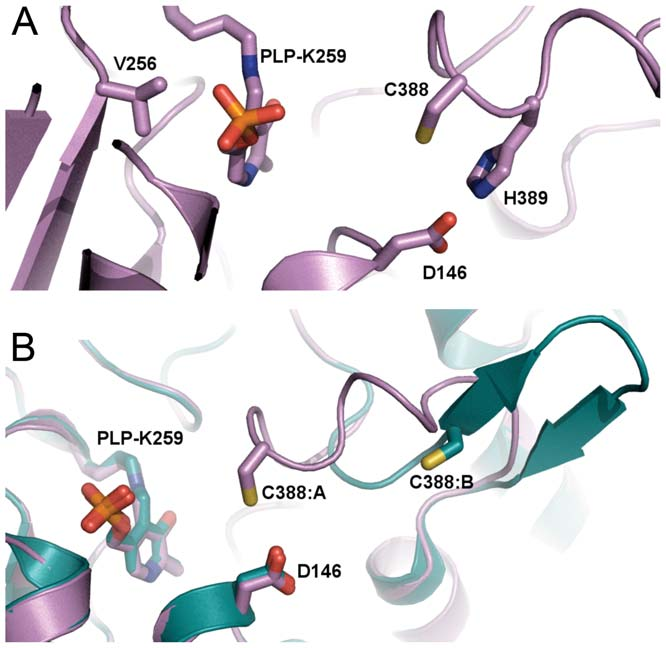
A) Position of residues D146K, V256S and H389T in relation to C388 and the PLP cofactor in the structure of hSCL (PDB id 3GZC). B) Superposed subunits A and B of human SCL (PDB id 3GZC) showing the structural differences in the active site segment and positioning of C388 (subunit A: pink “closed” and subunit B: cyan “open”), the location of Asp 146 is also shown.

**Figure 6:**
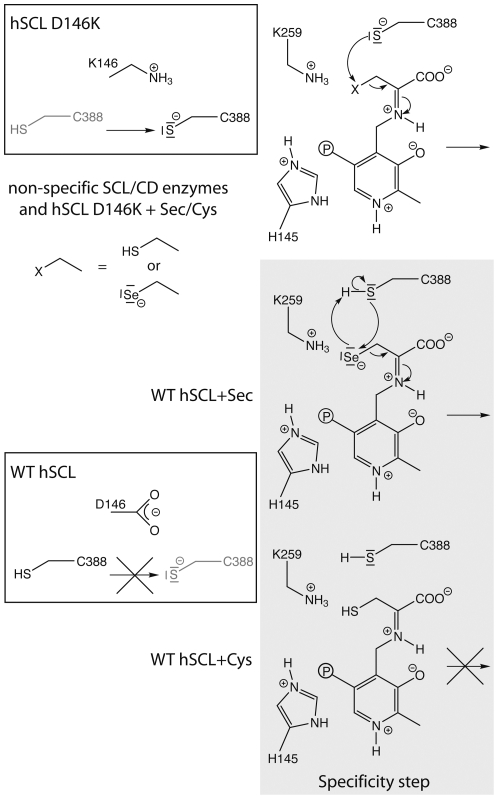
Proposed specificity step in hSCL shown with gray background, hSCL numbering. In non-specific enzymes the residue corresponding to 146 in hSCL is K in Group-I and H in Group-II SCL/CD proteins. The figure is drawn based on the mechanism involving elimination from the ketimine intermediate, originally proposed by Zheng et al. [16]. However, the same reasoning is equally valid also for the alternative mechanism with elimination directly from the quinonoid intermetiate (Fig. 1, red dotted arrows).

The crystal structure revealed that D146 interacts directly with C388 and is located on its opposite side in relation to the PLP cofactor and the substrate-binding site (Fig. 5A) [26]. This suggests that the effect of the D146K substitution is exerted through influencing C388 rather than affecting either the PLP cofactor or the substrate as such. A negatively charged Asp, as in the proteins specific for Sec, interacting with C388 should destabilize a deprotonated state while a positively charged Lys (as in other group I proteins) is expected to stabilize it (Fig. 6 boxes). In the group II CD/SCL proteins the corresponding residue is a His, which could serve as a base for hydrogen abstraction from the active site Cys and also stabilize the deprotonated form. Major differences between the two alternative substrates (Sec and Cys) must also be taken into account. With Sec acting as an “extreme Cys”, it should be noted that Se may be viewed as significantly more electrophilic than S [31], [32], while it is also clear that on the same time Sec is also more polarizable and nucleophilic than Cys [33] and a better leaving group more resistant to overoxidation [34]. Moreover, free Sec has a pKa of around 5.2 and is thus deprotonated at physiological conditions while for free Cys its pKa is around 8.3, leaving it mainly protonated. Even though Sec or Cys side chains within proteins may have significantly altered pKa values and that nucleophilicity/electrophilicity is not directly related to pKa [33], both the specific nucleophilicity/electrophilicity character and the large relative difference of ∼3 pH units may likely play a role for the substrate specificity of hSCL. For a nucleophilic attack of C388 to occur using Cys as an electrophilic substrate, the active site C388 residue needs to be deprotonated to form a Cys-persulfide [16], [17], [18], [19], [24], [30]. For Sec as substrate, on the other hand, it would be expected that the protonation state of C388 will be less critical, or even preferred to be in the protonated state, as the substrate itself is likely to be deprotonated and highly nucleophilic. In addition, a protonated C388 is clearly a better electrophile than a deprotonated C388, whereby the reaction would be expected to benefit from a more reactive substrate, such as Sec. Moreover, the completely conserved H145 and the positively charged ketimine nitrogen of the cofactor-substrate complex may be particularly effective to activate the Sec substrate because of the high polarizability of Se. Figure 6 illustrates the possible scenarios, for different substrates and the hSCL protein variants, in the step of the mechanism where C388 reacts with the substrate. Figure 6 is drawn based on the chemical mechanism involving elimination from the ketimine intermediate, originally proposed by Zheng et al. [16]. However, the same reasoning is equally valid also for the alternative shorter mechanism with elimination directly from the quinonoid intermetiate. Hence, we propose that Sec specificity over Cys occurs in hSCL because C388 is maintained in its protonated form, thus only reacting when Sec is bound to the PLP.

In addition, as described in the accompanying paper, group-I SCL/SD proteins contain a dynamic active site segment that houses the active site Cys residue. The location of D146 in relation to the dynamic active site segment [26] also appears ideally suited to impose a second level of control (Fig. 5B). In the closed conformation the sulfur atom of C388 is located <4 Å from D146, more or less in van der Waals contact, while in the open form this distance is ∼10 Å. A deprotonated, negatively charged, C388 is thus likely to shift the equilibrium towards the open or disordered state of the dynamic segment because of electrostatic repulsion from D146. This physico-mechanical mechanism is thus an additional and complementary way by which D146 may reduce the probability of positioning a deprotonated C388 in the closed conformation, as needed to react with Cys positioned into its substrate-binding cleft. Using the mechanisms described above as the basis for specificity should not yield a reaction that is strictly specific over infinite time scales because C388 would be deprotonated and located in the closed conformation a fraction of the time, depending on its local p*K*_a_, the pH, and the dynamics of the residue and active site domain. This is consistent with the observation of a C388-persulfide in the high-pH (P1) crystal form after a 2 h Cys soak [26] and the previously shown inhibition of the enzyme by Cys [9], [12], [13], [14], [15].

The results presented here are partially in contrast with the recent study of rSCL [25]. Instead of seeing an unproductive complex with Cys upon soaking as in rSCL (crystallized at pH 4.7 and soaked at pH 8.0), we observed the partial formation of a C388 persulfide after extended incubation of hSCL crystals with Cys (crystallized and soaked at pH 8.1) [26]. In addition, the stopped-flow data with hSCL and the variant protein with gained CD activity, shown herein, point to a chemical mechanism for the specificity in a step following initial substrate binding, rather than in the substrate binding itself. Still, it cannot be ruled out that several mechanisms work together or that there are mechanistic differences between hSCL and rSCL. In the in vivo scenario, where Cys may bind hSCL and thus block its active site and PLP cofactor availability for catalysis with Sec, other mechanisms e.g. direct protein-protein delivery facilitating Sec binding over Cys may also be in effect.

### Conclusions

Because of their similar chemical properties, the important discrimination between sulfur and selenium under physiological conditions has remained enigmatic. By studies of the Sec over Cys specificity of hSCL and the gain-of-function D146K/H389T protein variant, we here propose a possible mechanistic basis for a Sec-specific enzymatic reaction not accepting Cys as substrate. In this suggestion, the specificity is achieved by a combination of chemical and physico-mechanical control mechanisms, thereby exploiting the intrinsic chemical properties of the elements Se and S.

## Materials and Methods

Cloning, construction of protein variants, expression and purification was performed as described in the accompanying paper [26]. In short, the hSCL cDNA clone was purchased from the Mammalian Gene Collection (Genebank accession no. BC007891.2). The sequence encoding residues 8–445 was amplified by PCR and inserted into pNIC-Bsa4 vector (Genbank accession no. EF198106). The construct included an N-terminal His_6_ tag for purification. Variant SCLY constructs were made by using the Stratagene QuikChange® Multi Site-Directed Mutagenesis Kit using primers; D146K: 5′- cctcggtggaacac**a**a**g**tccatccggctgcc-3′ and H389T: 5′- ggggccgcgtgc**ac**ctcggaccacgg-3′, positions differing from wild-type are indicated in bold. After sequence confirmation, the wild-type and variant constructs were transformed into *E.coli* BL21(DE3)R3 pRARE cells and stored at −80°C as a glycerol stock. Cells from the glycerol stock were used to inoculate 20 ml of Terrific Broth (TB) supplemented with 8 g/l glycerol and 100 µg/ml kanamycin and grown at 30°C over night. The 20 ml culture was used to inoculate 1.5 l TB media supplemented with 8 g/l glycerol, 50 µg/ml kanamycin and 5 drops of BREOX anti-foaming agent (Cognis Performance Chemicals UK Ltd) in a 2 l glass flask. Cells were grown in a Large Scale Expression System (LEX, Harbinger Biotechnology) at 37°C until the OD_600_ reached 1. The cultivation was then cooled to 18°C for 1 h whereupon expression of hSCL was induced by the addition of 0.5 mM IPTG and subsequently continued over night at 18°C. Cells were harvested by centrifugation at 5500× g for 10 min. at 4°C and the pellet was resuspended in lysis buffer containing 50 mM Na-phosphate pH 7.5, 500 mM NaCl, 10% glycerol, 10 mM imidazole, 0.5 mM TCEP, and Complete EDTA-free protease inhibitor (Roche Biosciences). The sample was sonicated on ice (Sonics VibraCell) at 80% amplitude, 4 sec on, 4 sec off for a total of 3 min followed by centrifugation at 49 000× g for 20 min. at 4°C. The soluble fraction was decanted and filtered through a 0.45 µm filter. Purification of the protein was performed as a two-step process on an ÄKTAxpress system (GE Helthcare). First step, metal affinity chromatography using 1 ml HiTrap Chelating column (GE Helthcare) and second step, gel filtration using, Superdex 200 gel filtration column (HiLoad 16/60; GE Healthcare). Prior to purification, the HiTrap Chelating column was equilibrated with *buffer1* (50 mM Na-phosphate pH 7.5, 500 mM NaCl, 10% glycerol, 10 mM imidazole, 0.5 mM TCEP) and the Superdex 200 was equilibrated with *buffer2* (20 mM Hepes pH 7.5, 300 mM NaCl, 10% glycerol, 0.5 mM TCEP. The filtered lysate was loaded onto the Ni-charged HiTrap Chelating column and washed with *buffer1* followed by *buffer1* supplemented with imidazole to a final concentration of 25 mM. Bound protein was eluted from the column with *buffer1* containing 500 mM imidazole and automatically loaded onto the gel filtration column, and subsequently eluted using *buffer2*. Fresh TCEP was added to the pooled fractions to a final concentration of 2 mM. Protein identity was confirmed by mass spectrometry. Prior to spectroscopic measurements, the His-tag was cleaved off and removed by IMAC chromatography. Before assaying, the protein was treated with EDTA, which was subsequently removed by dialysis against *buffer2*.

*Time-resolved spectroscopy-* The reaction between SCL and cysteine (Cys) was monitored using a stopped-flow spectrophotometer (Applied Photophysics, Leatherhead, UK). A solution containing 20 mM Cys, 120 mM Tris pH 8.5 and 2 mM TCEP was rapidly mixed at a ratio of 1∶1 with a solution containing 40 µM of enzyme, 120 mM Tris pH 8.5 and 2 mM TCEP. Absorbance changes as a function of time were monitored either at several wavelengths simultaneously using a diode-array detector or at single wavelengths using a photomultiplier tube. The absorbance at time = 0 was set to zero independently of the actual value, which means that the absolute absorbance values at different wavelengths can not be directly compared.
